# Effect of Surface Treatment on Shear Bond Strength between Resin Cement and Ce-TZP/Al_2_O_3_

**DOI:** 10.1155/2016/7576942

**Published:** 2016-06-13

**Authors:** Jong-Eun Kim, Jee-Hwan Kim, June-Sung Shim, Byoung-Duck Roh, Yooseok Shin

**Affiliations:** ^1^Department of Prosthodontics, College of Dentistry, Yonsei University, Seoul 03722, Republic of Korea; ^2^Department of Conservative Dentistry, Oral Science Research Center, College of Dentistry, Yonsei University, Seoul 03722, Republic of Korea

## Abstract

*Purpose.* Although several studies evaluating the mechanical properties of Ce-TZP/Al_2_O_3_ have been published, to date, no study has been published investigating the bonding protocol between Ce-TZP/Al_2_O_3_ and resin cement. The aim of this study was to evaluate the shear bond strength to air-abraded Ce-TZP/Al_2_O_3_ when primers and two different cement types were used.* Materials and Methods.* Two types of zirconia (Y-TZP and Ce-TZP/Al_2_O_3_) specimens were further divided into four subgroups according to primer application and the cement used. Shear bond strength was measured after water storage for 3 days or 5,000 times thermocycling for artificial aging.* Results.* The Y-TZP block showed significantly higher shear bond strength than the Ce-TZP/Al_2_O_3_ block generally. Primer application promoted high bond strength and less effect on bond strength reduction after thermocycling, regardless of the type of cement, zirconia block, or aging time.* Conclusions.* Depending on the type of the primer or resin cement used after air-abrasion, different wettability of the zirconia surface can be observed. Application of primer affected the values of shear bond strength after the thermocycling procedure. In the case of using the same bonding protocol, Y-TZP could obtain significantly higher bond strength compared with Ce-TZP/Al_2_O_3_.

## 1. Introduction

Metal-ceramic restorations are among the most commonly used treatment options in fixed prosthodontics. However, their drawbacks include discoloration due to the metal frame showing at the gingival margin, opaque shade unlike natural teeth, and possible metal allergy [1].

The interest in zirconia as a dental restorative material is rapidly increasing. Zirconia has biocompatibility and excellent mechanical properties such as high fracture resistance. It also has shading similar to natural teeth, and it is possible to fabricate natural-looking esthetic restorations. In particular, with the recent advances in CAD/CAM systems, restorations with ceramic build-up on a zirconia framework and restorations composed totally of zirconia are commonly used in daily dental practice. For these reasons, zirconia is receiving a spotlight as a nonmetal restoration material that could replace metal-ceramic restorations [2–7].

Yttrium-stabilized tetragonal zirconia (Y-TZP) is a polycrystalline ceramic with three crystal phases: monoclinic, tetragonal, and cubic. Yttrium oxide controls the volume expansion and stabilizes the crystal such that the tetragonal phase can be maintained at room temperature [5, 8]. The characteristic that distinguishes zirconia from other ceramics is that when a crack initiates and propagates because of external forces, the tetragonal phase around the crack can transform to monoclinic phase. Such transformation causes 3–5% volume expansion, and the compressive stress layer around the crack may stop the spread of the crack [7, 9, 10]. However, one of the drawbacks is that, in the oral environment where the material is consistently exposed to moisture, low temperature degradation occurs. This effect deteriorates the mechanical properties of zirconia [11, 12].

Zirconia is chemically and biologically inert, but it is difficult to obtain predictable bonding in clinical dental use [4, 6, 7]. For glass ceramics, etching with hydrofluoric acid is the standard protocol, but zirconia is resistant to acid-etching as it contains less glass and is mainly composed of crystal [13–15]. Consequently, micromechanical bonding through air-abrasion, roughening by diamond rotary instruments, and chemical bonding through primer application have been recommended [7, 16, 17]. Roughening the zirconia surface through airborne abrasion with 50 *μ*m Al_2_O_3_ particles increases the shear bond strength of resin cement [18, 19]. Strategies to roughen the surface and obtain chemical bonding have led to the development of primer and luting cement. Various resin bond strength studies with the application of methylacryloyloxydecyl dihydrogenphosphate (MDP) monomers after airborne abrasion were performed, and such protocols have been used clinically. In prosthodontics, MDP-containing resin cement is also used [7].

Cerium oxide can also be used for tetragonal phase stabilization [10, 20]. Recently a Ce-TZP/Al_2_O_3_ nanocomposite that contains alumina has been developed. Alumina was interpenetrated with zirconia to enhance the strength of the Ce-TZP/Al_2_O_3_ nanocomposite, and it consists of 10 mol% Ce-TZP and 30 vol% Al_2_O_3_ [11, 21, 22]. Equally distributed Al_2_O_3_ improves the hardness, elastic modulus, and stability in moist environments [23, 24]. The Ce-TZP/Al_2_O_3_ nanocomposite has higher fracture resistance compared to conventional Y-TZP. While Y-TZP consists of homogenous grain, the Ce-TZP/Al_2_O_3_ nanocomposite has an interpenetrated intergranular nanostructure. Nanometer-sized Al_2_O_3_ particles are located in submicron-level ZrO_2_ grains, and the ZrO_2_ particles are located in Al_2_O_3_ grains. This structure improves the strength of Ce-TZP/Al_2_O_3_ [25, 26]. In addition, it is more resistant to low temperature degradation (LTD) in a moist environment compared to Y-TZP and has shown satisfactory results in terms of phase transformation and mechanical properties [21].

While there have been many studies regarding adhesion to zirconia and there have been reports on the mechanical properties of Ce-TZP/Al_2_O_3_, studies on adhesion of Ce-TZP/Al_2_O_3_ have been scarce. The purpose of this study was to compare the shear bond strength to air-abraded Y-TZP and Ce-TZP/Al_2_O_3_ when primers and two different cement types were used.

## 2. Materials and Methods 

### 2.1. Zirconia Specimen Fabrication and Surface Treatment

**Figure 1:**
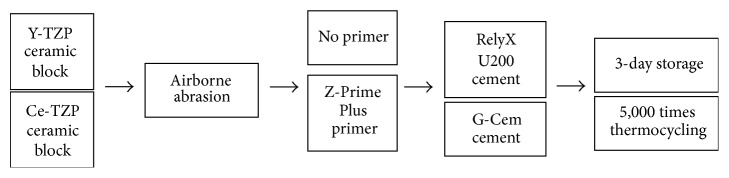
Flowchart of the zirconia block surface treatments.

Zirconia discs were sectioned to fabricate specimens after a sintering process. The specimens were embedded in polyethylene molds with cold curing resin (Vertex Dental, Singapore). One side of the disc was exposed and polished with 600-grit silicon carbide paper under water irrigation. After polishing, the blocks were put in an ultrasonic water-bath filled with tap water. The surface treatment method is described in detail in Figure 1, and the materials used in the study are shown in Table 1. Airborne abrasion was performed with 50 *μ*m sized Al_2_O_3_ particles with 3.5-bar pressure at 10 mm distance for 15 seconds. Afterwards, additional surface treatment was performed according to the experimental group, and cementation was performed with resin cement.

The zirconia specimens were divided into two groups: Y-TZP (Katana Zirconia; Noritake Dental Supply Co., Ltd., Japan) and Ce-TZP/Al_2_O_3_ (NanoZR; Panasonic Health Care, Japan). All specimen surfaces were treated with airborne abrasion, and they were further divided into four subgroups according to primer application and the cement used: (1) group NG: cemented with G-Cem (GC Corp., Japan) without any primer application; (2) group NU: cemented with RelyX U200 (3M ESPE, Seefeld, Germany) without any primer application; (3) group PG: cemented with G-Cem linkace cement after Z-Prime Plus primer (Bisco Inc., Schaumburg, IL) application; (4) group PU: cemented with RelyX U200 after Z-Prime Plus primer application.

One hundred sixty specimens in total were divided into 16 groups of 10 specimens each. Primer and resin cement application were performed according to the manufacturer's instruction, and resin cement was applied to a plastic mold (Ultradent Jig; Ultradent Products, South Jordan, USA) to place the resin cement on the zirconia surface (bonding area 4.45 mm^2^). The plastic mold containing resin composite cement was placed on the zirconia surface and light-cured with a 1200 mW LED light curing unit (DB-686 Cappu LED Curing Light; Bisco Asia, Seoul, Korea). After light curing from 4 different directions for 20 seconds each, 80 specimens were left to further polymerize at room temperature (23 ± 1°C) for one hour and then kept in a water bath containing tap water at 37°C for 3 days. The other 80 specimens were thermally cycled with 5,000 cycles between 5°C and 55°C and a dwell time of 30 seconds.

### 2.2. Shear Bond Strength Test

Shear bond strength was measured using a shear bond tester (Bisco Inc., Schaumburg, IL, USA). The crosshead speed was set at 0.5 mm/min. The force was applied to the adhesion surface of the specimen, and the maximum fracture strength was measured in N. The measuring data were converted into MPa by dividing by the bonding area.

### 2.3. SEM Analysis

**Figure 2:**
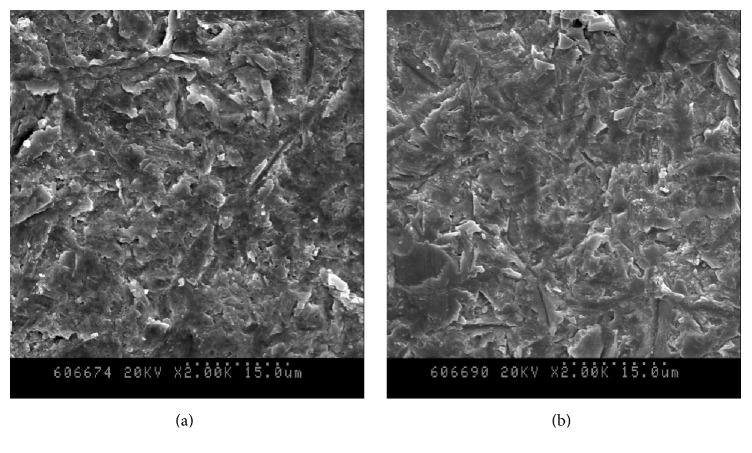
SEM of the surface of the specimens after air-abrasion with 3.5-bar pressure: (a) Ce-TZP/Al_2_O_3_ block; (b) Y-TZP block.

The surface of the specimens after air-abrasion with 3.5-bar pressure was analyzed by scanning electron microscopy (SEM) (Figure 2). The zirconia blocks were mounted after carbon adhesive application and coated with gold-palladium. Representative images were obtained from each group at 2,000x magnification.

### 2.4. Failure Mode Analysis

The fractured specimen interface was analyzed with an optical microscope using 10.0x magnification. The failure mode was classified as one of the following: (1) adhesive failure: failure at the interface between zirconia and resin cement; (2) cohesive failure: failure within resin; (3) mixed failure: combination of adhesive and cohesive failure.

### 2.5. Statistical Analysis

Statistical analysis was performed with SPSS v23.0 (SPSS Inc., Chicago, IL, USA) software. Three-way ANOVA analysis was performed to see the influence of the following factors: aging time, type of zirconia block, and surface treatments and their interaction on mean SBS (*p* < 0.05). The data were separated with respect to aging time and type of zirconia block for statistical analysis by one-way ANOVA with regard to the surface treatments used followed by a post hoc Bonferroni test (*p* < 0.05).

## 3. Results

### 3.1. Shear Bond Strength of Resin Cement to Y-TZP and Ce-TZP/Al_2_O_3_ Ceramic

The mean bond strength values according to different zirconia blocks, bonding materials, and aging time are shown in Table 2. Three-way ANOVA showed that the type of zirconia block, bonding material, and aging time have a significant effect on shear bond strength (*p* < 0.05). A significant interaction between aging time and bonding material used was observed. However, significant interactions between other factors were not observed: aging time and type of zirconia block (*p* > 0.05), bonding material and type of zirconia block (*p* > 0.05), and aging time × type of zirconia block × bonding material (*p* > 0.05).

When the same surface treatment method and cement were used, there was a significant difference between the Y-TZP and Ce-TZP/Al_2_O_3_ blocks. The Y-TZP block showed significantly higher shear bond strength than the Ce-TZP/Al_2_O_3_ block generally. In the same zirconia block and aging time, G-Cem linkace cement showed higher bond strength than RelyX U200 cement with no priming. However, after primer application, RelyX U200 showed higher bond strength compared to G-Cem linkace cement.

After a thermocycling procedure, the bond strength values of specimens using G-Cem linkace cement were decreased significantly. On the other hand, the bond strength values of RelyX U200 specimens were increased. Primer application promoted high bond strength and less effect on bond strength reduction after thermocycling regardless of type of cement, zirconia block, or aging time.

**Figure 3:**
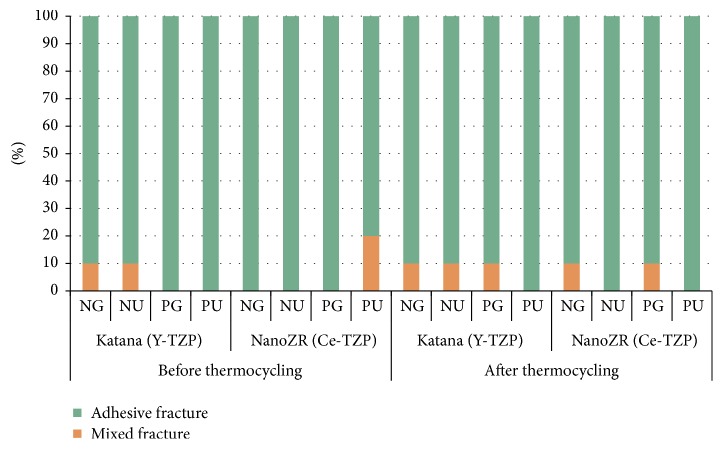
The failure modes of shear bond tests before and after thermocycling. NG (no primer and G-Cem), NU (no primer and RelyX U200), PG (Z-Prime Plus primer and G-Cem), and PU (Z-Prime Plus primer and RelyX U200).

Most specimens showed adhesive failure and mixed failure, with adhesive failure occurring in the highest proportion. Only a small number of specimens showed mixed failure. In the Ce-TZP block, the PU group where RelyX U200 was used with primer application showed the highest percentage of mixed failure before thermocycling. No significant association was observed between the failure patterns and the shear bond strength (Figure 3).

## 4. Discussion

This study evaluated the shear bond strength of resin cement to Y-TZP and Ce-TZP/Al_2_O_3_ depending on whether a primer and/or thermocycling procedure is applied. The importance of air-abrasion in order to achieve a stable and high bond strength to zirconia surfaces has been shown in a previous study [27], and consequently that protocol was included in this study. Air-abrasion increases the surface area and wettability of oxide ceramics such as zirconia by making the surface rougher and consequently improves the bond strength to resin cement. Air-abrasion also removes organic contaminants [27].

Z-Prime Plus contains phosphate monomer and carboxylate monomer. The phosphate monomer bonds with metal oxides such as zirconia [4]. Carboxylate groups in Z-Prime Plus reinforce the adhesion to zirconia. When a primer including phosphorylated monomer is applied to zirconia, the bond strength is improved, and the primer also acts as a wetting agent for the resin cement.

The two resin cement types used in the current study are self-adhesive resin cement, which do not require the use of additional adhesives or primers, thereby saving time and effort. In general, self-adhesive resin cement is known to show weaker bond strength compared to self-etching resin cement or total etching resin cement [28]. However, because it contain MDP, it reacts chemically with metal oxide and shows relatively high bond strength [29].

This study showed that the shear bond strength of the NG groups is high, and group NU has low bond strength for 3-day specimens. There seems to be a difference in filler content and viscosity between the two cement types. G-Cem linkace cement was not significantly influenced by the application of primer unlike RelyX U200. When G-Cem linkace cement was used for Y-TZP cementation, there was not any difference in bond strength depending on primer application. On the other hand, the bond strength of RelyX U200 cement significantly improved after primer application. When primer was applied, there was a significant difference in bond strength according to the different resin cement types. Because RelyX U200 resin cement has higher filler content and viscosity, primer application to zirconia increased its wettability and allowed easier penetration of the resin cement [30]. Another study also reported that primer application dramatically increases the bond strength when RelyX U200 is used [31]. This is consistent with previous studies [27, 32] and shows that both air-abrasion and primer application contribute to effective bonding. On the other hand, contradictory results on primer application can be found. A previous study reported that primer application did not have any effect on bond strength to air-abraded Y-TZP surfaces [29]. In other studies, the bond strength significantly improved after air-abrasion with 50-*μ*m Al_2_O_3_ particles, and air-abrasion was a more significant factor than primer application [33, 34].

G-Cem linkace cement received more impact from thermocycling. Although whole groups using G-Cem linkace cement showed high shear bond strength in the 3-day groups, it was decreased after the thermocycling procedure. The primer treated group exhibited a slight decrease, and the nonpriming group showed a sharp decrease of bond strength. In contrast, there was a slightly different aspect in the groups using RelyX U200 cement. Group PU of the Y-TZP block demonstrated a significantly high elevation of bond strength after 5,000 times thermocycling. Although there was no significant difference, the Ce-TZP/Al_2_O_3_ block showed a slight increase of bond strength. Group NU specimens did not exhibit a significant difference between the 3-day and 5,000 times thermocycling groups. As opposed to the groups using G-Cem linkace cement that showed reduced bond strength, it was a different pattern.

This aspect has been reported in several previous papers. It is known that RelyX U200 has moisture tolerance and contributes by complete polymerization of the chemically cured part during thermocycling [35].

In the comparison of the type of zirconia blocks, the Y-TZP block has higher bond strength compared with the Ce-TZP/Al_2_O_3_ block. Through the SEM pictures, it was difficult to find significant differences between the two blocks on the surface of the specimens having undergone air-abrasion to 3.5 bar; it is considered that there are close associations with a difference in composition. In the case of Ce-TZP/Al_2_O_3_, not only does it consist of mere zirconia, but also it forms a nanocomposite with Al_2_O_3_ for optimal fracture strength. So, there is some possibility that the differences of bond strength were influenced by the material composition. The possibility exists of an interaction between the Ce-TZP/Al_2_O_3_ nanocomposite and the powder for air-abrasion including Al_2_O_3_ as well. Ce-TZP/Al_2_O_3_ is known to be more sensitive to stress-induced transformation [36], and the transformation after air-abrasion probably changed the surface and affected the bond strength. The necessity for more in-depth scientific research may be considered in the field of Ce-TZP/Al_2_O_3_ materials itself.

In this study, 3.5-bar pressure was used for air-abrasion using previous studies as [37–39]. Hallmann et al. [40] reported that as the pressure of air-abrasion increased, greater surface changes occurred and the surface became rougher. A rougher surface results in more microretentive area and allows for mechanical interlocking with the resin cement. In the SEM image, intergrain space is observed in the abraded zirconia surface [14]. Air-abrasion treatment forms more hydroxyl groups on the ceramic surface and enhances the chemical reaction with MDP [33].

Several studies have reported that air-abrasion with high pressure damages the zirconia surface and deteriorates its mechanical properties. In order to minimize surface damage, air-abrasion with low pressure should be used, and a phosphate monomer containing primer is needed for a durable bond [41, 42]. Yang et al. [29] reported that air-abrasion with 2.5-bar pressure increases the surface roughness, increases the wettability of resin cement without additional primer application, and allows MDP within the resin cement to contribute to high bond strength. Conversely, if abrasion is performed with a low pressure of 0.5 bar and primer is not applied, significantly lower bond strength was achieved. If MDP-containing primer is used after the same low pressure air-abrasion treatment, significantly higher bond strength was obtained. The primer application compensated for the difference in surface roughness due to the different air-abrasion pressure. According to these results, if low pressure air-abrasion is used or resin cement without functional groups is used, primer application is significant for achieving high bond strength. As reported by Hallmann et al. [40], air-abrasion may increase the chance of surface cracks or surface damage despite increasing the roughness. Therefore, it may clinically worsen the prognosis of zirconia restoration

For further evaluation, additional experiments with greater numbers of specimens are needed. Because adhesion studies on Ce-TZP/Al_2_O_3_ are scarce, further studies to find the optimal adhesion protocol depending on the pressure used for air-abrasion and primer application are needed. In addition, the phase transformation of Ce-TZP/Al_2_O_3_ after air-abrasion and the consequent change in fracture resistance and bond strength should be investigated.

## 5. Conclusions

Depending on the type of the primer or resin cement used after air-abrasion, different wettability on zirconia surfaces can be observed. Application of primer affected the values of shear bond strength after a thermocycling procedure. In the case of using the same bonding protocol, the Y-TZP block could obtain higher bond strength compared with the Ce-TZP/Al_2_O_3_ block.

## Figures and Tables

**Table 1:** Experimental materials used in this study.

Material	Brand	Manufacturer
Zirconia	Katana Zirconia (Y-TZP)	Noritake Dental Supply Co., Ltd., Japan
	NanoZR (Ce-TZP/Al_2_O_3_)	Panasonic Health Care, Japan

Primer	Z-Prime Plus	Bisco Inc., Schaumburg, IL

Resin cement	RelyX U200 cement	3M ESPE, USA
	G-Cem linkace cement	GC Corp., Japan

**Table 2:** Median shear bond strength in MPa to zirconia surfaces before and after thermocycling (*p* < 0.05).

Thermocycling	Zirconia block	No primer		Z-Prime Plus	
		G-Cem	RelyX U200	G-Cem	RelyX U200
Before	Katana (Y-TZP)	30.24^A^_a_^α^	14.71^C^_a_^α^	25.21^AB^_a_^α^	22.56^B^_a_^β^
	NanoZR (Ce-TZP/Al_2_O_3_)	22.89^A^_b_^α^	14.24^B^_a_^α^	20.48^AB^_b_^α^	24.59^A^_a_^α^

After	Katana (Y-TZP)	19.09^B^_a_^β^	16.46^B^_a_^α^	23.60^AB^_a_^α^	29.69^A^_a_^α^
	NanoZR (Ce-TZP/Al_2_O_3_)	14.12^B^_a_^β^	12.27^B^_a_^α^	16.15^B^_b_^β^	26.02^A^_a_^α^

Note: statistical differences are indicated by different superscript upper case letters (within a column for the same zirconia block and ageing process) or by different subscript lower case letters (within a row for the same surface treatment and aging process). Letters *αβ* in superscript reflect significant differences within a row for assessing effects of thermocycling within the same zirconia block.
